# *Ex vivo* 2D and 3D HSV-2 infection model using human normal vaginal epithelial cells

**DOI:** 10.18632/oncotarget.14840

**Published:** 2017-01-27

**Authors:** Yaqi Zhu, Yan Yang, Juanjuan Guo, Ying Dai, Lina Ye, Jianbin Qiu, Zhihong Zeng, Xiaoting Wu, Yanmei Xing, Xiang Long, Xufeng Wu, Lin Ye, Shubin Wang, Hui Li

**Affiliations:** ^1^ State Key Laboratory of Virology, Institute of Medical Virology, Wuhan University School of Basic Medical Sciences, Wuhan, Hubei 430071, China; ^2^ Shenzhen R and D Center of State Key Laboratory of Virology, Wuhan University Shenzhen Institute, Shenzhen, Guangdong 518057, China; ^3^ Xiangyang No.1 People's Hospital, Xiangyang, Hubei 441000, China; ^4^ Peking University Shenzhen Hospital, Shenzhen, Guangdong 518036; ^5^ Hubei Maternal and Child Health Hospital, Wuhan, Hubei 430070, China; ^6^ Shenzhen Eye Hospital, Shenzhen, Guangdong 518040, China

**Keywords:** three dimension (3D), ex vivo, human normal vaginal epithelial cells (HNVEC), air-liquid interface (ALI) culture, HSV-2 infection model

## Abstract

Herpes simplex virus type 2 (HSV-2) infects human genital mucosa and establishes life-long latent infection. It is unmet need to establish a human cell-based microphysiological system for virus biology and anti-viral drug discovery. One of barriers is lacking of culture system of normal epithelial cells *in vitro* over decades. In this study, we established human normal vaginal epithelial cell (HNVEC) culture using co-culture system. HNVEC cells were then propagated rapidly and stably in a defined culture condition. HNVEC cells exhibited a normal diploid karyotype and formed the well-defined and polarized spheres in matrigel three-dimension (3D) culture, while malignant cells (HeLa) formed disorganized and nonpolar solid spheres. HNVEC cells had a normal cellular response to DNA damage and had no transforming property using soft agar assays. HNVEC expressed epithelial marker cytokeratin 14 (CK14) and p63, but not cytokeratin 18 (CK18). Next, we reconstructed HNVEC-derived 3D vaginal epithelium using air-liquid interface (ALI) culture. This 3D vaginal epithelium has the basal and apical layers with expression of epithelial markers as its originated human vaginal tissue. Finally, we established an HSV-2 infection model based on the reconstructed 3D vaginal epithelium. After inoculation of HSV-2 (G strain) at apical layer of the reconstructed 3D vaginal epithelium, we observed obvious pathological effects gradually spreading from the apical layer to basal layer with expression of a viral protein. Thus, we established an *ex vivo* 2D and 3D HSV-2 infection model that can be used for HSV-2 virology and anti-viral drug discovery.

## INTRODUCTION

Herpes Simplex Virus type 2 (HSV-2) is one of the most common genital pathogens and prevalent sexually transmitted viruses. The latest global estimates for 2012 of the burden of prevalent (existing) and incident (new) HSV-2 infection are 417 million and 19.2 million people, respectively [1]. The primary site of HSV-2 infection is genital tract mucosal epithelium and viruses subsequently replicate within vaginal keratinocytes [2]. The damaged ulcerative genital tract mucosa provide opportunities for other detrimental pathogens to readily invade the vagina [3]. HSV-2 infection not only establishes a lifelong asymptomatic latency in the neurons of sensory ganglia but also can be periodically reactivated and lead clinical or subclinical palindromia. The viral reactivation and ulcer disease by genital HSV-2 infection may increase 5-fold potential risk of HIV-1 transmitted efficiency and in an up-regulation of HIV replication through trans-activating the long terminal repeat (LTR) of HIV [4–6]. HSV-2 has recently been reported to be a risk factor for Kaposi's sarcoma [7]. At present clinical anti-viral therapies mainly target the process of HSV-2 reactivation but not eliminate latent viruses [8]. Currently, there is no effective prophylactic or therapeutic vaccine available for genital HSV-2 infection [9]. One of the major reasons is lacking of virus research system which is close to human physiological conditions *in vitro*.

The genital mucosa is not only the first physical line of defense against HSV-2 infection but also involves in orchestrating innate immunity and adaptive immunity [10]. Genital epithelium is layered and polarized epithelial tissue which shapes two distinct surfaces for apical and basolateral domains. The morphology and the degree of differentiation of epithelial tissues play an important role in viral immune response. The replication of HSV-2 in genital mucosal epithelium and the continued spread to nervous system have largely depended on the polarized epithelium [11]. The conventional two dimensional (2D) culture has obvious limitations as viral infection model. There also exist inherent differences between human and animal. The anti-HSV-2 vaccines tested on traditional animal models are hardly applied to clinic treatments [4]. Therefore, it is of great importance to establish a human cell-based microphysiological system for virus biology research.

Recently researchers have gained their attentions to this point. A Canadian group established an *ex vivo* culture model and investigated the susceptibility of primary human female genital epithelial cells to HSV-2 [10]. They also assessed the anti-viral activity of human female genital epithelium in response to HSV-2 and the role of HSV-2 virion host shutoff protein on dsRNA antiviral pathways in human vaginal epithelial cells [12]. Another report demonstrated that HSV-2 infection induces CXCL9 expression in primary cervical epithelial cells and recruits activated CD4(+) T cells to mucosal HSV-2 infection sites and potentially increases the risk of HIV-1 sexual transmission [13]. We also established immortalized human cervical epithelial (HCE) cells *in vitro* model and demonstrated that TLR4 plays a critical role in innate immune response to HSV-2 infection [14–16]. However, human normal tissue-derived primary cells will undergo senescence after very limited passages *in vitro*, resulting in a poor stability and repeatability of experiments. The immortalized cells by exogenous genes transduction often change their genetic background and phenotype [17]. Therefore, these immortalized cells lost the normal physiological functions [18].

In the current study, we isolated the primary human normal vaginal epithelial cells (HNVEC) by irradiated feeder co-culture technology [18]. The primary cells propagate stably and rapidly *in vitro* and retain the normal biological characteristics. Most importantly, we successfully reconstructed the polarized vaginal epithelium using the three-dimensional (3D) air-liquid interface (ALI) culture and verified its morphological features identical to the originated vaginal tissue. Furthermore, we established a novel HSV-2 infection model with 3D ALI cultures. This *ex vivo* 3D viral infection model possesses the susceptibility to HSV-2. We observed the replication of virus and viral pathological effects in a time-depended manner. This 3D HSV-2 infection model may provide a human cell-based microphysiological system more close to the natural infection process of HSV-2 for virus biology research and anti-viral drug discovery.

## RESULTS

### Isolation and propagation of human normal vaginal epithelial cells (HNVEC)

**Figure 1:**
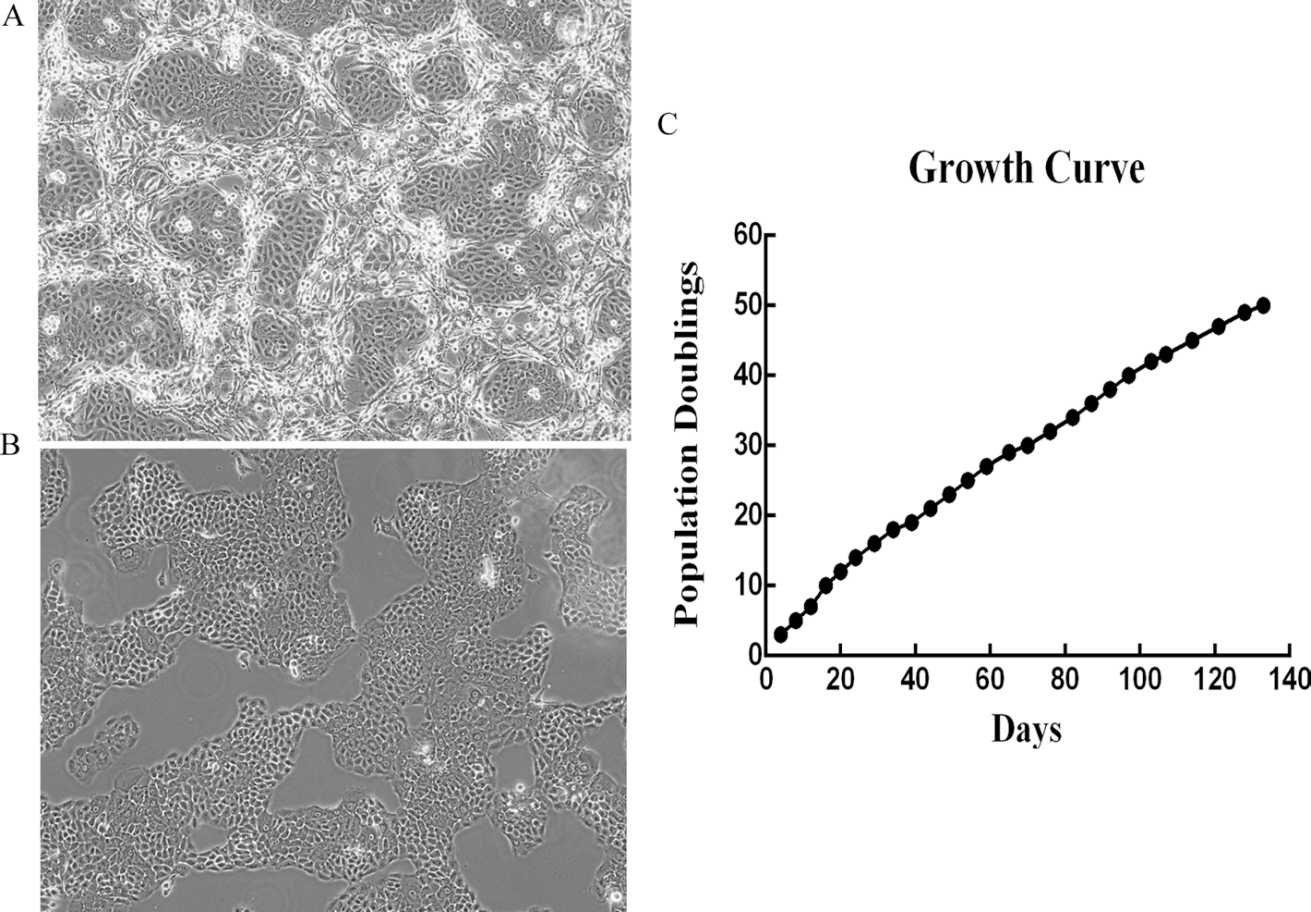
The isolation and propagation of human normal vaginal epithelial cells (HNVEC) (**A** and **B**) The morphology of the primary HNVEC cells. The vaginal tissue was harvested and digested with trypsin and collagenase, then cultured with feeder cells (A) and in primary epithelial culture medium (B) as described in Materials and Methods. HNVEC cells propagated rapidly and morphology was photographed on day 7 under the phase contrast microscope. Magnification 10×. (**C**) The growth curve of HNVEC cells. The numbers of HNVEC cells were counted for each passage, and a plot of accumulated population doublings versus growth days was constructed.

The vaginal tissues were digested and dispersed into the single cells as described in Materials and Methods. Initial culture was established with irradiated feeder fibroblasts. After 2 days of plating, small colonies were readily observed. Then epithelial cells were cultured in a defined medium as described in Materials and Methods. HNVEC cells proliferated rapidly to reach confluence in approximately 5 to 6 days. Images of HNVEC cells co-cultured with feeder cells and in primary epithelial culture medium (PECM) were shown in Figure 1A and 1B, respectively. The Short Tandem Repeats (STR) analysis (DNA fingerprinting) was performed in order to confirm the uniqueness of HNVEC. HNVEC cells have 21 STR loci and a couple of X-chromosome-specific Amelogenin loci (Supplementary Figure 1A). STR analysis verified that HNVEC cells were originated from a specific individual and do not match any other cell lines published or registered in the database of ATCC, DSMZ, JCRB and RIKEN. HNVEC cells proliferated rapidly and the cell numbers were recorded at each passage. The growth curve of HNVEC cells was plotted as accumulative population doublings versus days. Figure 1C showed a constant growth of HNVEC cells with 50 accumulated population doublings for 133 days. Telomerase reverse transcriptase (TERT) is the active subunit of telomerase which maintains the telomere's length during cell division. Usually the hTERT expression is turned off in most somatic cells, while tumor-derived cell lines have reactivated hTERT expression. Telomerase plays a critical role in primary cell immortalization and hTERT expression was induced in conditionally reprogrammed primary normal epithelial cells [18, 19]. To analyze the hTERT activation, total RNAs of HNVEC cells were harvested at passage 4, 14, 24, 34 and the expression of hTERT was detected by quantitative RT-PCR as described in Materials and Methods. The expression of hTERT was induced at the very early passage (p4) in HNVEC cells and declined thereafter (Supplementary Figure 1B). The tendency was maintained stably at late passages (p14, p24, p34). As a control, the cancer cells (HeLa) have a much higher expression of hTERT. The results demonstrated that HNVEC cells sustained a continuous and stable proliferation status during the period of passaging.

### HNVEC cells maintain normal karyotypes and response to DNA damage and non-oncogenic

**Figure 2:**
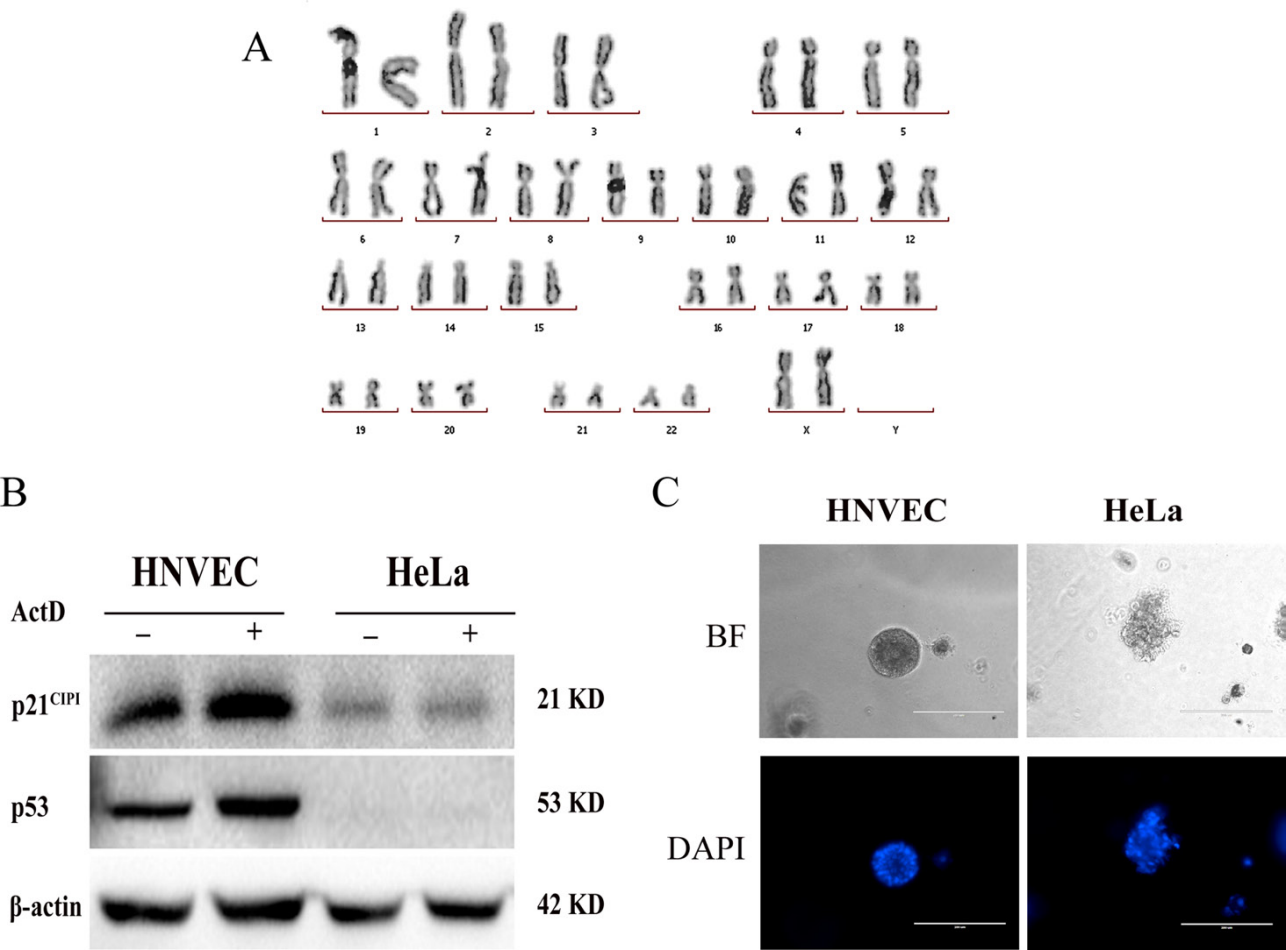
HNVEC cells maintain normal karyotype and response to DNA damage (**A**) Normal karyotype of HNVEC cells. Twenty metaphase spreads were analyzed and photographed under the microscope. The representative normal karyotype (46, XX) of cells was shown. (**B**) Normal response to DNA damage. DNA damage was induced by treatment of cells with 0.5 nM Actinomycin D (ActD) for 24 hours. The response was measured by the levels of p53 protein and its downstream target p21 by western blotting assay. Actin is a loading control. The experiment was repeated three times, the representative data were shown. (**C**) Differential potential of HNVEC cells. Single-cell suspension of HNVEC and HeLa cells were cultured in medium containing 2% matrigel for 7 days. The morphology of cell aggregates was stained by 0.5 μg/ml DAPI and photographed by fluorescence microscopy. Scale bar, 200 μm.

The histology of the mirrored structure of specimen confirmed no contamination of tumor lesions in the normal vaginal tissues (Supplementary Figure 2A). Next, we analyzed whether HNVEC cells harbored any abnormal karyotype by the conventional cytogenetic analysis. Figure 2A demonstrated that HNVEC cells possess a structurally and numerically normal karyotype with a 46,XX. The tumor suppressor gene p53 effectively arrests the aberrant cells suffered from genetic damage and prevents abnormal proliferation. The expression of p53 is increased if normal cells experienced gene damage. The increased expression of p53 activates the activity of downstream effector p21^CIP1^. The up-regulated expression of p21^CIP1^ is able to immediately arrest cell cycle at G1 phase and restraint malignant propagation [19, 20]. In most exogenous genes (such as hTERT or HPV16-E6/E7) induced immortalized cells or cancer-derived cell lines (such as HeLa cells), the p53 signal pathways generally exhibit mutant or suppressed status. To verify whether HNVEC have the intact p53-mediated growth related pathways and normal function to respond to the DNA damage, HNVECs were treated with a low dose of actinomycin D (Act D) for 24 hrs, HeLa cells were used as the control. Act D triggers nucleolar disruption [21] and induces DNA strand breakage, leading to p53-dependent cell growth arrest [19]. As shown in Figure 2B, p53 protein level was up-regulated in HNVECs with ActD treatment and the downstream effector p21^CIP1^was also increased compared to untreated HNVEC cells. However, in HeLa cells treated with Act D, p53 protein level was not upregulated and neither p21^CIP1^was induced compared to untreated HeLa cells. Thus, HNVEC cells possess a normal response to DNA damage. The differential potential of cells is one of cellular hallmarks of normal physiological function. To evaluate the differential potential of HNVEC cells, we performed matrigel 3D cultures. Matrigel basement membrane matrix, a kind of extracellular matrix (ECM) rich in laminin, is a critical regulatory factor to retain normal homeostasis and tissue morphology [22, 23]. As relevant important signal pathways are usually lost in traditional two-dimensional (2D) culture using plastic substrata, matrigel 3D culture was able to discriminate between benign or malignant mammary cells [24]. Figure 2C showed that HNVEC cells formed well-defined and polarized spheres in this 3D context, while HeLa cells (a cervical cancer cell line) generated proliferous, disorganized and nonpolar solid aggregates. Thus, HNVEC cells have a normal function to differentiate in matrigel 3D culture. We also evaluated the transforming property of HNVEC cells by soft agar assay. Since anchorage-independent growth is a representative feature of tumor cells *in vitro* proliferation. As shown in Supplememtary Figure 2B, HeLa cells readily grew as scobinate and anchorage-independent colonies. In contrast, HNVEC cells did not form cell colonies and existed in the form of single cells or cell debris in soft agar culture for 30 days. These data indicated that HNVEC cells have normal biological characteristics.

### HNVEC cells express CK14 and p63, not CK18

**Figure 3:**
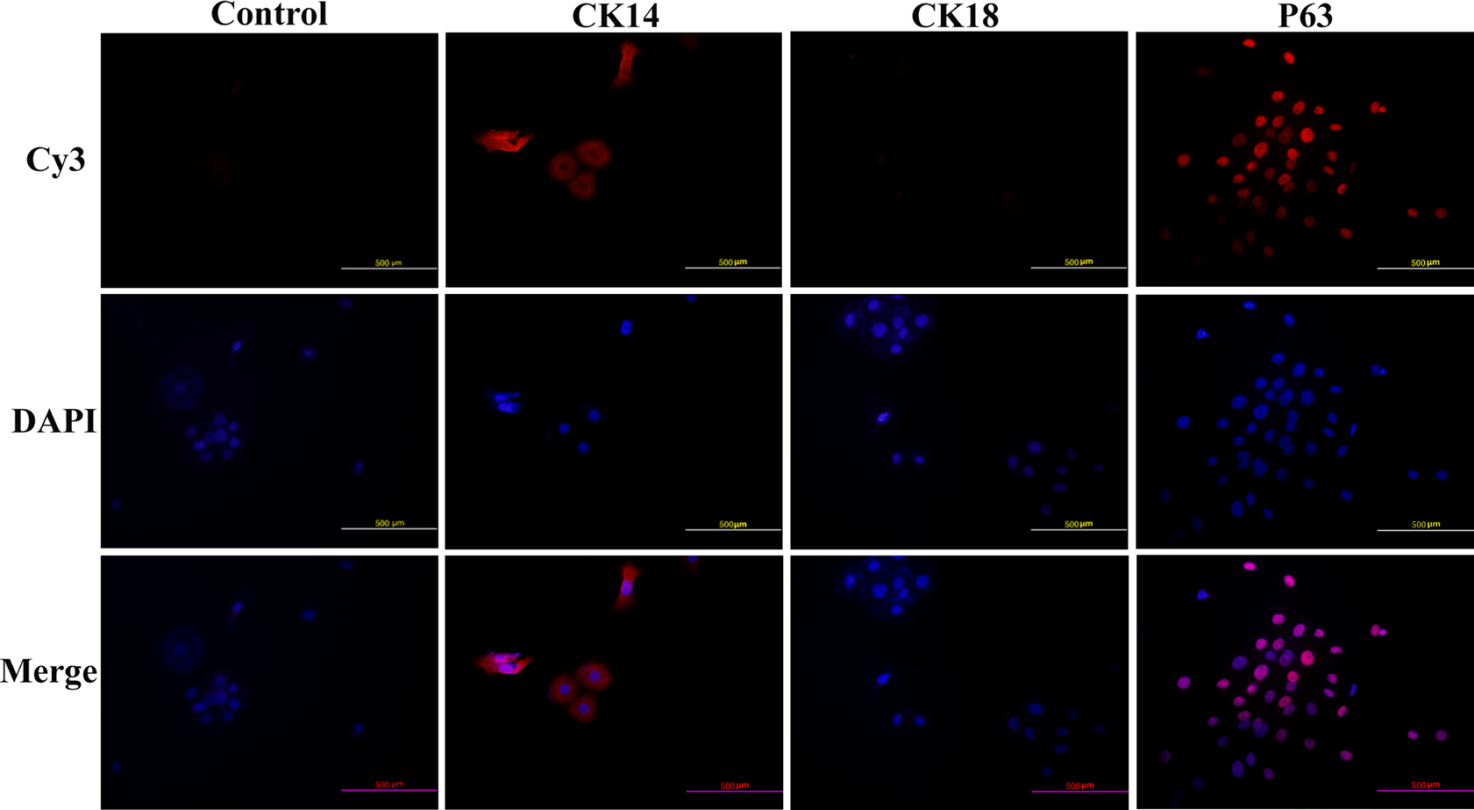
HNVEC cells express CK14, not CK18 The HNVEC cells were cultured on the sterile glass cover slips at an appropriate density and fixed in 4% (w/v) paraformaldehyde, permeabilized with 0.5% Triton-X-100 and labeled with the primary antibodies against CK14, CK18 and p63 respectively. These three protein makers were detected by immunofluorescence assay. The nuclei were stained by 0.5 μg/ml DAPI. The proteins were stained by second antibody goat-anti-mouse IgG—Cy3. Scale bar, 500 μm.

The normal cervical epithelium contains two obviously morphological types: one is non-keratinized squamous epithelium lining the ectocervix (the vaginal portion of the cervix); and the other is columnar mucus-secreting epithelium attaching to the endocervical canal and its corresponding glands. The vaginal wall tissues, affiliated to ectocervix, are rich in vaginal epithelial cells which belong to stratified squamous epithelium without keratinized cell layers. The cytokeratins expression of ectocervix in stratified squamous epithelium immediately above the basal layers displayed positive for CK14. In columnar endocervix as well as in cervical adenocarcinoma, CK18 was observed positive while CK14 negative [25]. Therefore, the immunofluorescence assay was performed to examine the expression of CK14 and CK18 of HNVEC cells. As shown in Figure 3, the cytokeratin expression of normal HNVEC cells was positive for CK14 and negative for CK18. This data verified that HNVEC cells were originated from the stratified squamous epithelium of ectocervix. Since conditionally reprogrammed cells represent a stem-like state and possess the ability of indefinite proliferation *in vitro* [26]. We also detected the expression of p63 which is an essential transcription factor for morphological development and stemness maintenance of stratified epithelial structures [27]. The expression of p63 was readily observed in the nuclei of HNVEC cells by immunofluorescence (Figure 3).

### HNVEC-derived three-dimensional (3D) cultures are similar to the original human vaginal tissue

**Figure 4:**
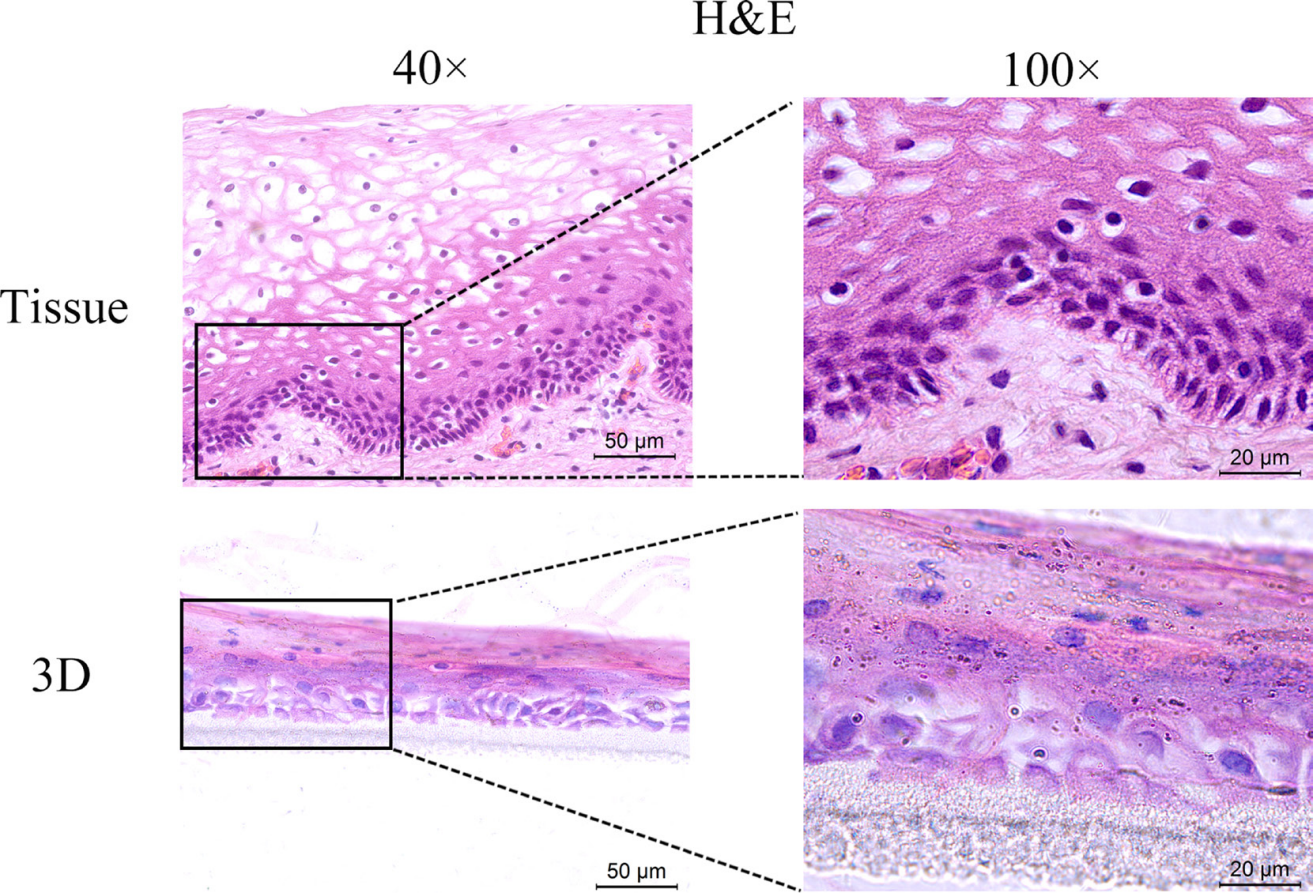
H&E-staining of the original vaginal tissue and 3D cultures The HNVEC cells were cultured in air-liquid interface (ALI) for 14 days before fixed. The originated human vaginal tissue or 3D cultures were fixed by 4% paraformaldehyde (w/v), and then paraffin-embedded and sectioned using standard histological procedures. The result of H&E staining was photographed under the microscope. Scale bar, 50 μm and 20 μm.

**Figure 5:**
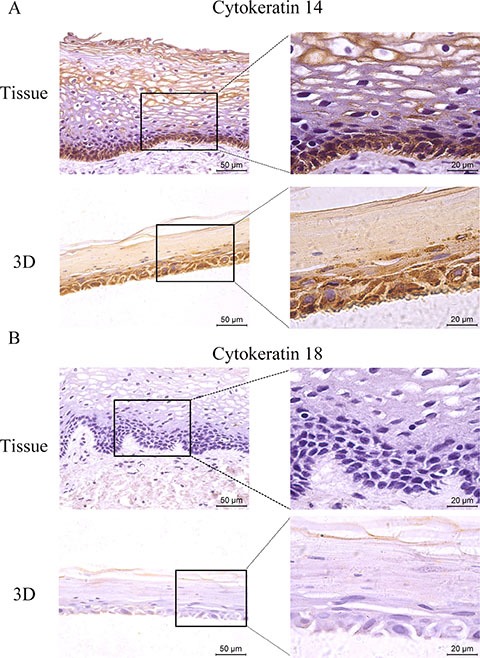
The expression of CK 14 and 18 The HNVEC cells were cultured in air-liquid interface (ALI) for 14 days before fixed. The originated human vaginal tissue or 3D cultures were fixed by 4% paraformaldehyde (w/v), and paraffin-embedded, sectioned, detected by immunohistochemical staining with the specific antibodies against CK 14 (A) or CK 18 (B) . Scale bar, 50 μm and 20 μm.

**Figure 6:**
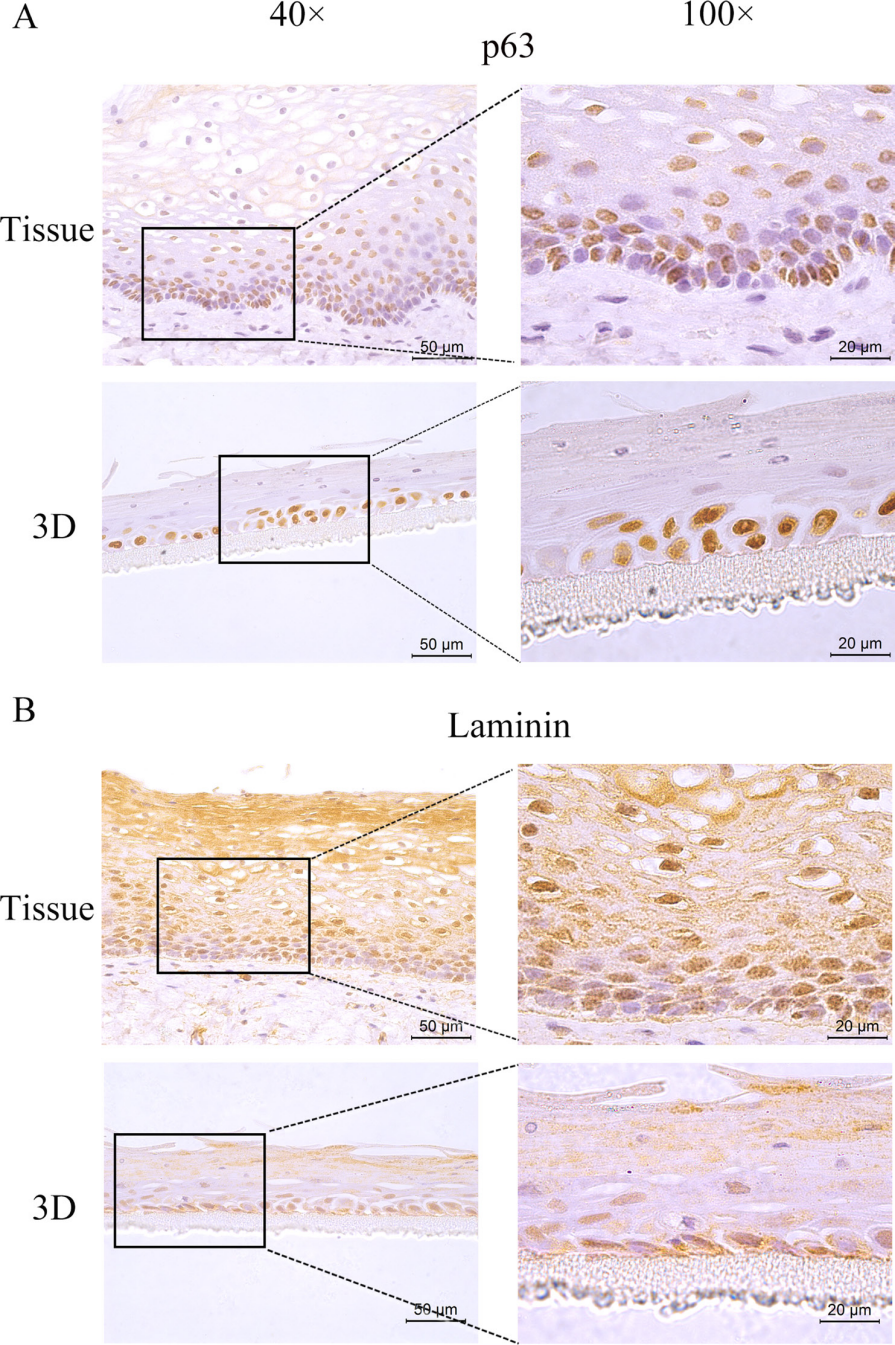
The expression of p63 and Laminin The HNVEC cells were cultured in air-liquid interface (ALI) for 14 days before fixed. The originated human vaginal tissue or 3D cultures were fixed by 4% paraformaldehyde (w/v), and paraffin-embedded, sectioned, detected by immunohistochemical staining with the specific antibodies against p63 (**A**) or Laminin (**B**). Scale bar, 50 μm and 20 μm.

**Figure 7:**
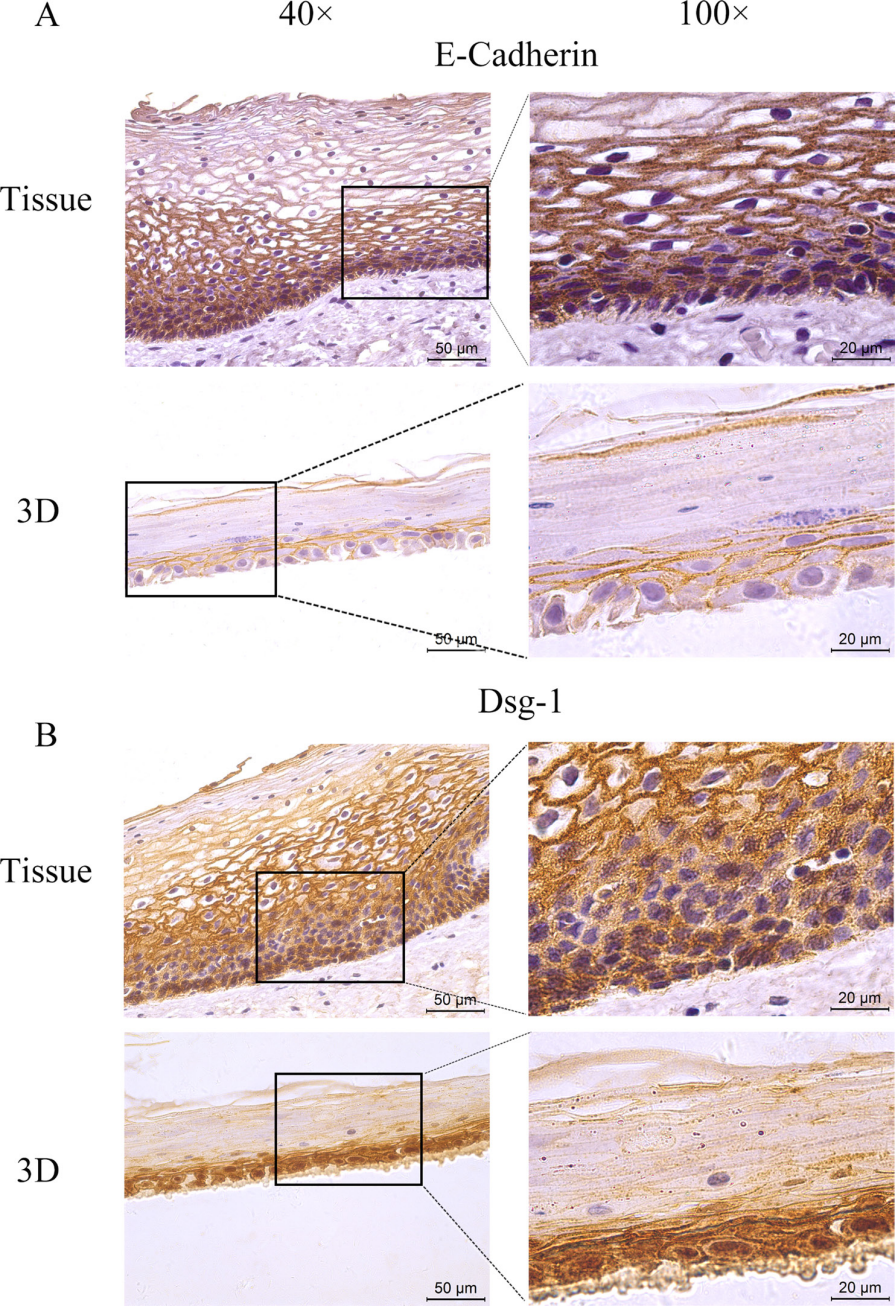
The expression of tight junction markers The HNVEC cells were cultured in air-liquid interface (ALI) for 14 days before fixed. The originated human vaginal tissue or 3D cultures were fixed by 4% paraformaldehyde (w/v), and paraffin-embedded, sectioned, detected by immunohistochemical staining with the specific antibodies against E-cadherin (**A**) or DSG-1 (**B**). Scale bar, 50 μm and 20 μm.

Genital epithelium is layered and polarized epithelial tissue which shapes two distinct surfaces for apical and basolateral domains [28]. The above data showed that HNVEC cells have the normal biological features and differentiated potential. Therefore, we reconstructed the polarized vaginal epithelium using 3D air-liquid interface (ALI) culture as described in Materials and Method. We compared the epithelial structures between the originated vaginal tissue and constructed 3D culture by H&E staining (Figure 4). HNVEC-derived 3D culture displayed the typical stratified epithelium with polarity similar to its originated vaginal tissue. The basal epithelial cell layers with stemness proliferation, intermediate transformation zone and superficial keratinized cell layers were distinctly observed. The data indicated that HNVEC cells establish a well-differentiated squamous epithelium with polarization under a stress force of differentiation. To further verify whether reconstructed 3D cultured vaginal epithelia have the identical epithelial specificity to the original vaginal tissue, six markers (CK14, CK18, p63, Laminin, E-cadherin and Dsg-1) were detected using immunohistochemical assay (Figure 5, Figure 6 and Figure 7). Desmoglein-1 (Dsg-1) plays a pivotal role in compensating for tight junctions in multiple epithelial tissues [29] and normally expresses in the human epidermis, throughout all nucleate cell layers [30]. Cell-to-cell adhesion, a typical characteristic of human epithelium, is mediated by a vital calcium-dependent receptor called Epithelial-cadherin (E-cadherin) [31]. E-cadherin-mediated adhesion greatly contributes to maintaining the integrity of epithelium in homeostasis of internal micro-environment, and balancing the communication between intracellular junction and cell proliferation [32]. The expression pattern in the original vaginal tissue was: p63 expressed in the nuclei of basal epithelial cell layers (Figure 6); CK14 (Figure 5) and Laminin (Figure 6) expressed in the cytoplasm of basal layers, E-cadherin and Dsg-1 (Figure 7) expressed both in the cytoplasm of basal layers and transformation zone (the expression level of Dsg-1 is higher than that of E-cadherin); CK18 (Figure 5) not expressed in all cell layers compared with negative control (Supplementary Figure 3) . Reconstructed 3D culture showed the similar expression pattern as the corresponding vaginal tissues. Taken together, the reconstructed 3D cultures represent an *ex vivo* model which mimics the histological structure of vaginal epithelium in morphology and physiologic function.

### Establishment of reconstructed 3D vaginal epithelium HSV-2 infection model

**Figure 8:**
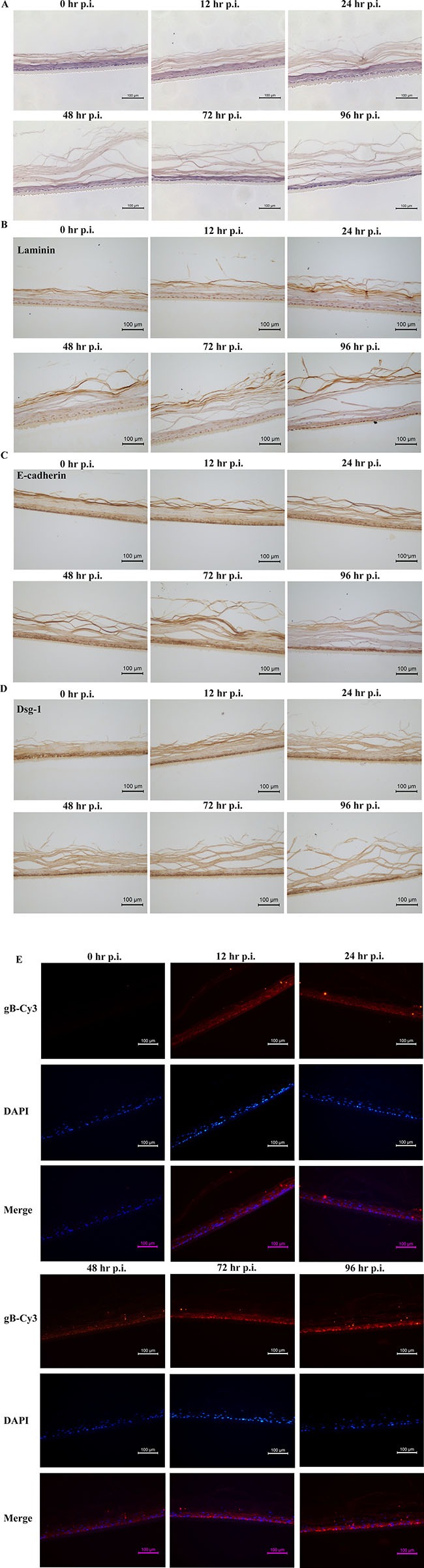
HSV-2 infection on ALI 3D cultures (**A**) Morphology of reconstructed 3D vaginal epithelium HSV-2 infection model. The ALI 3D cultures were harvested and fixed by 4% paraformaldehyde (w/v) at the indicated time points at 0, 12, 24, 48, 72, and 96 hrs after HSV-2 infection. The 3D culture at 0 hrs p.i. was set as the control. The fixed cultures were then incubated overnight at 4°C and paraffin-embedded, sectioned using standard histological procedures. The results of H&E stain were photographed under the microscope. Magnification 20×. Scale bar, 100 μm. (**B**) Expression of Laminin. The above ALI 3D cultures infected with HSV-2 at indicated time points were detected by immunohistochemical staining with antibody against Laminin. Magnification 20×. Scale bar, 100 μm. (**C**) Expression of E-Cadherin. The above ALI 3D cultures infected with HSV-2 at indicated time points were detected by immunohistochemical staining with antibody against E-Cadherin. Magnification 20×. Scale bar, 100 μm. (**D**) Expression of Dsg-1. The above ALI 3D cultures infected with HSV-2 at indicated time points were detected by immunohistochemical staining with antibody against Dsg-1. Magnification 20×. Scale bar, 100 μm. (**E**) Expression of HSV-2 gB protein. The above ALI 3D cultures infected with HSV-2 at indicated time points were labeled with primary antibody gB and the nuclei were stained by 0.5 μg/ml DAPI. The gB protein were then stained by second antibody goat-anti-mouse IgG-Cy3. The gB expression was photographed under the fluorescence microscope. Magnification 20×. Scale bar, 100 μm.

On the basis of reconstructed normal HNVEC-derived 3D model, we established a HSV-2 infected vaginal epithelium 3D model. The ALI 3D cultures were inoculated with HSV-2 (G strain, 4 × 10^5^ PFU/well) at the apical layers after HNVEC ALI cultures maintained for 12 days. At the indicated time points, 0, 12, 24, 48, 72, and 96 hrs p.i., the 3D cultures were collected and fixed. The morphological alterations of HNVEC-derived 3D model after HSV-2 entry were examined by H&E staining (Figure 8A). At 0 hrs p.i., 3D vaginal cultures formed a normal stratified squamous epithelium structure and intact basal cell layers with compact-arranged keratinized epidermis. As early as 12 hrs after HSV-2 infection, the keratinized epidermis became loosed. The keratinized layers of the apical stratified epithelium became gradually incompact and detached upon 24 hrs and 48 hrs p.i.. After 72 hrs of viral infection, most of the keratinized layers of apical surface were lost and the loose appearance reached the basal layers. Even the basal cell layers were damaged and became very thin at 96 hrs p.i. (Figure 8A). Meanwhile, the observation that the interior of 3D cultures tuned wet (medium permeated from bottom of the inserts) indicated the damaged air-liquid interface environment. Therefore, we detected the expression of Laminin and two other epithelial tight junction markers, E-cadherin and Dsg-1, by immunohistochemical assay (Figure 8B, 8C, 8D). The results showed that the integrity of 3D vaginal epithelium tight junction was being destroyed gradually from the apical surface to basal layers as the time went on after HSV-2 infection. The pathological changes of 3D cultures throughout all the cell layers in vaginal epidermis were clearly observed.

Glycoprotein B (gB), a late expression protein of herpes simplex virus (HSV), is widely considered to be a highly conserved envelop glycoprotein. gB protein is most abundant in HSV which can specifically recognize and bind to an appropriate receptor on the membranes of the host cells to initiate the membrane fusion process [33]. HSV-2 enters into the host cells through with a fusion of either plasma or endosomal membrane with the help of this recognition. Therefore, we investigated the expression of gB protein in the reconstituted vaginal 3D epithelium by immunofluorescence assay. The gB protein was not expressed in 3D culture at 0 hrs p.i. (Figure 8E). As early as 12 hrs post infection, gB protein was obviously observed on account of transcription of viral protein after HSV-2 absorption and penetration into the host cells. The expression level of gB protein was slightly decreased at 24 hrs p.i. till 48 hrs p.i.. And then the level of gB protein expression increased at 72 hrs p.i. and reached the peak at 96 hrs p.i. (Figure 8E). Along with the progression of viral infection and lytic release of the virions, HSV-2 proliferated and replicated in the reconstructed 3D vaginal epithelium and destroyed the epithelial cell layers as well as cell-cell tight junctions. The obvious pathological effects gradually spread from the apical layer to basal layer in a time-depended manner. This reconstituted 3D viral infection model manifested the susceptibility to HSV-2 invasion and the pattern of internal virus infection. These results are expected to provide an *ex vivo* microphysiological system more close to the situation of natural infection of HSV-2.

## DISCUSSION

HSV-2 infection is mainly implicated in epithelial cells [34]. The latent-period viruses are prone to be periodically reactivated and lead to life-threatening recurrent diseases [35]. Currently treatments with anti-viral therapy do not eliminate latent viruses. There is no effective preventative or therapeutic vaccine and drug available for genital HSV-2 infection [9]. It is of great importance to establish a human cell-based micro-physiological system for further HSV-2 biology research and anti-viral drug discovery.

*In vitro* culture of normal epithelial cells has been a big challenge over decades. The normal cells experience two critical phases during proliferation: senescence (M1 phase) and crisis (M2 phase) [36]. The transduction of exogenous genes, for example HPV E6/E7, can make normal cells span both M1 and M2 phases and be immortalized [18, 36]. Genetic manipulations frequently resulted in the changes of genetic backgrounds, the failures of normally physiological functions, as well as the inhibitions of some important signal pathways [18].

Animal models are also mostly used as the research tools to develop anti-viral drugs or vaccines. In the past five years, numerous promising prophylactic or therapeutic anti-HSV-2 vaccines are tested on animal models, the most frequently used are mice and guinea pigs [37]. The inherent differences between human and other mammalians always hinder the applications of animal models to human diseases. The viral research system which is close to human physiological conditions is still highly desirable.

Most recently, conditionally reprogrammed cells technology provides a solution [18]. This technique can efficiently establish normal epithelial cell cultures from different mammalian tissues by using fibroblast feeder cells and a Rho kinase inhibitor, without transduction of exogenous genes. These reprogrammed cells can proliferate indefinitely and rapidly *in vitro* and retain a normal karyotype and non-tumorigenicity. The most important finding is that the induction of the reprogrammed cells is reversible, removal of Rho kinase inhibitor and feeders allows the cells to differentiate normally [26]. Reprogrammed cells may represent an adult stem-like state of epithelial cells. The activation of telomerase, remodeling of cytoskeleton and interference of p16/Rb signal pathways have been speculated to be the critical and indispensable prerequisites [18, 19].

We successfully established human normal vaginal epithelial cells (HNVEC) from human vaginal tissue through reprogrammed cells technology. They can propagate rapidly *in vitro* and retain the normal biological characteristics. The short tandem repeat (STR) analysis exhibited that the HNVEC cell line was newly generated from a specific individual without matching any others database published or registered before (Supplementary Figure 1A). Matrigel 3D culture and nucleus staining showed that HNVEC cells formed well-defined and smooth spheres [22, 38].

Since the normal functional epithelium has physiological polarity *in vivo* and form two distinct surfaces according to their different distribution of transmembrane proteins and lipids: the apical domain and the basolateral domain. The former mainly faces to the outside atmosphere and pernicious pathogens, and the latter contacts with the bottom cells and systemic vasculature [28]. The polarized structure of epithelia play a significant role in the invasion and release of the virus which perhaps alternatively infect either the apical domain or the basal domain [39]. For instance, the SV40 viruses are inclined to infect the bodies from apical domain [40], while the VSV and Semliki Forest virus are apt to invade from basal domain [41, 42]. So, 2D culture system cannot satisfy our demands for further exploration of viral researches. 3D culture, a widely-utilized cell culture system *in vitro*, can be employed to visualize the cellular basis of morphogenesis in epithelial cells and develop the currently popular individual-based treatments [43–45]. The polarized epithelium 3D models recapture the growth and differentiation *in vivo* environment and overcome the species-discrepant limitations of animals, therefore representing a fire-new *ex vivo* model for viral biological research.

In this study, the air-liquid interface (ALI) culture has been applied to reconstitute the polarized and stratified vaginal epithelium 3D model. HNVEC-derived 3D culture displayed the typical stratified epithelium with polarity similar to its originated vaginal tissue (Figure 4). They commonly expressed the specific keratin marker CK14 (Figure 5), stemness-maintained marker p63 (Figure 6) and cell-to-cell tight junction proteins E-cadherin and Dsg-1 (Figure 7). More importantly, we established a HSV-2 infected vaginal epithelium 3D model on the basis of reconstructed normal HNVEC-derived 3D model. The pathological changes of 3D cultures after HSV-2 entry throughout all the cell layers were clearly observed (Figure 8A). The expression of two epithelial tight junction markers, Dsg-1 and E-cadherin, showed that the integrity of 3D vaginal epithelium was being destroyed gradually from the apical surface to basal layers as the time went on after HSV-2 infection (Figure 8C and 8D). The expression of viral protein, gB, demonstrated that the progression of HSV-2 proliferation and replication in the reconstructed 3D vaginal epithelium in a time-dependent manner (Figure 8E). This reconstituted 3D viral infection model manifested the susceptibility to HSV-2 invasion and the pattern of internal virus infection.

The HNVEC cells generated using reprogrammed cell technique and HNVEC-based 3D vaginal epithelium are originated from the individual patient without any genetic manipulation. The reprogrammed HNVEC cells possess the normal biological characteristics and reconstituted 3D vaginal epithelium mimics the physiological *in vivo* status. The combination of this 2D and 3D human cell-based cultures will be a valuable and novel models for viral biological research and anti-viral drug discovery.

## MATERIALS AND METHODS

### Cell isolation

With the written informed consent of the patient, adjacent normal vaginal tissues from a surgical specimen of vaginal tumor was collected. Institutional review boards of Wuhan University Shenzhen institute and Hubei Maternal and Child Health Hospital approved this study. The normal tissue was obtained as far from the tumor lesion as possible to avoid contamination of tumor tissue for the specimen. In addition, mirror structure of specimen was prepared for histological examination. Uncontaminated specimen was immediately put into the fresh clinical sample preservation medium and sent to research lab. The mirror structure of specimen was separated and performed histology experiments. The other half of specimen was minced and dispersed into the single cells with dispase plus collagenase (Stem Cell Technologies Inc, Vancouver, Canada, BC). The primary vaginal epithelial cells were cultured according to co-culture protocol with irradiated feeder cells and cells were then propagated in primary epithelial culture basic medium (PECBM, ImmorTech, Shenzhen, China). The recipe of primary epithelial culture medium was described in early study [46]. The cells were cultured and proliferated at 37°C in a humidified incubator, with 5% CO_2_. The cell growth curve was plotted as accumulated population doublings versus time (days)[19].

### STR analysis

Cellular genome DNA of primary HNVEC cells was extracted using a commercial kit (Tiangen, Beijing, China). Short tandem repeat (STR) analysis (DNA fingerprinting) was performed by commercial kit (PowerPlex21 System; Promega Corporation, Madison, WI). This system recognizes the co-amplification and three-color detection of 22 loci (21 STR loci and the X-chromosome-specific Amelogenin). The PCR amplification was achieved on the basis of the manufacturer's recommended protocol with the ABI 3100 genetic analyzer (Thermo Fisher Scientific, Massachusetts, USA). The following STR markers were detected except to the Amelogenin loci: Amel, CSF1PO, D10S1248, D12S391, D13S317, D16S539, D18S51, D19S433, D21S11, D2S1338, D2S411, D3S1358, D5S818, D6S1043, D7S820, D8S1179, FGA, Penta D, Penta E, TH01, TPOX, vWA. Allele size determination and related data analysis were accomplished with Genotyper and Power Typer 21 Macro Software (Thermo Fisher Scientific, Massachusetts, USA).

### Karyotype analysis (conventional cytogenetic analysis)

The exponentially growth HNVEC cells were treated with 0.08 μg/ml colchicine for 3 hrs and observed to be a tendency of cytomorphosis and cell rounding. Cells were then collected and digested into 0.05% trypsin, continued with a hypotonic treatment using 0.075 mol/L KCL and fixation in the mixture of methanol and glacial acetic acid. Mitosis metaphase spreads were stained with Giemsa dye and imaged under the optical microscope. Approximately twenty metaphase spreads were calculated the numbers of chromosomes and analyzed their morphology under the microscope (BX51TF, Olympus Company, Tokyo, Japan).

### Matrigel three-dimensional (3D) culture

Single-cell suspensions of HNVEC and HeLa cells were used and prepared into a specifically differential medium (keratinocyte growth medium, Life Technologies Corporation, California, USA) containing 5% pre-cooling Matri-gel (Discovery Labware, Inc., Two Oak Park, Bedford, MA). Matri-gel presents a liquid state at 4°C, while solid phase at 37°C. The matrigel cultures were incubated at 37°C in a humidified incubator, with 5% CO_2_. Morphogenesis assays (DAPI staining) were performed after 7 days culture as previously described [22, 47]

### Air-liquid interface (ALI) culture

Millicell PCF inserts (12mm size, Millipore, Massachusetts, USA) were placed into a 6-well plate and made them completely wet with PECBM (ImmorTech, Shenzhen, China)before inoculating cells. Single-cell suspensions of HNVEC with PECBM were prepared to add into the each insert. About 2 ml PECBM was also dropped into the well (outside the inserts). The 6-well plate was then put in an incubator (37°C, 5% CO_2_, humidity) and cultured about 48 hrs. PECBM was totally replaced with differentiation medium (CELLnTEC Advanced Cell Systems AG, Switzerland) inside and outside the inserts and incubated for 16 hrs to allow cells to form an intercellular adhesion structure. The fresh differentiation medium was changed every 2 or 3 days. The 3D cultures were differentiated approximately 14 days and then fixed with 4% paraformaldehyde to perform histology experiments.

### Virus infection

Herpes simplex virus 2 (HSV-2,G strain) was obtained from American Type Culture Collection (ATCC). All stocks of HSV-2 used in this study were propagated in Africa green monkey kidney (Vero) cells. The titer of virus stocks was determined by a standard plaque assay and titers were generally expressed with plaque-forming units (PFU)/ml. The HSV-2 stocks were usually frozen and stored at −80°C in 1ml aliquots so as to ensure that every aliquot of virus stocks was fresh for each experiment. Vero cells were proliferated in complete Dulbecco's modified Eagle's medium (DMEM) supplemented with 8% fetal bovine serum (FBS) and 1% penicillin/streptomycin. For virus infection, vaginal epithelium 3D culture was infected with HSV-2 of 4 × 10^5^ PFU/well. The virus inoculums were removed after 2 hrs adsorption and fresh differentiation medium (CELLnTEC Advanced Cell Systems AG, Switzerland) was added. The 3D culture was continued cultured in 37°C incubator until collected at the indicated time points.

### Western blotting analysis

HNVEC cells were added with or without a low dose of 0.5 nM actinomycin D (Act D) for 24 hrs and then scratched using a cell scraper into 200μl modified radioimmuno-precipitation assay (RIPA) buffer containing protease inhibitors as described in early study [15] with HeLa cells as a control. The collected protein samples were measured concentration by BCA (Beyotime Biotechnology, Shanghai, China) method using microplate spectrophotometer (Bio-Tek, Vermont, USA). SDS polyacrylamide gels electrophoresis was conducted and then transferred electrophoretically on to a 0.2μm polyvinylidenedifluoride (PVDF) membranes (Immobilon-NC, Millipore, Italy), specifically probed with following primary antibodies: mouse anti-p53 (1:1000, Santa Cruz Biotechnology, CA, USA, sc-126), rabbit anti-p21 (1:1000, Santa Cruz Biotechnology, CA, USA, sc-397) and mouse anti-β-actin (1:100, Proteintech Group, Inc., Chicago, USA) 4°C overnight and then conjugated with the following secondary antibodies: HRP-labeled goat anti-mouse/rabbit lgG (Santa Cruz Biotechnology, CA, USA). Immunoblots were colorated with an mixture of enhanced chemiluminescence reagents ECL A and B (Beyotime Biotechnology, Shanghai, China) at a ratio of 1:1 and digitally photographed in UV trans-illuminator (Bio-rad Laboratories, California, USA).

### Immunofluorescence assay

The HNVEC cells were cultured on the sterile glass coverslips to an appropriate density and fixed in 4% (w/v) paraformaldehyde, permeabilized with 0.5% Triton-X-100 and labeled the primary antibodies (1:100, mouse anti-CK14, Santa Cruz Biotechnologies, CA, sc-23878; 1:100, mouse anti-CK18, Santa Cruz Biotechnologies, CA, sc-32329; 1:100, mouse anti-p63, Abcam, Cambridge, UK, ab735) and the secondary antibodies (1:100, fluorescently-labeled goat anti-mouse lgG-cy3, BA1031, Boster company, Wuhan, China) according to the manufacture's protocol. The vaginal tissues and paraffin sections of 3D cultures were solvent-dewaxed and treated with microwave antigen retrieval. The primary antibodies (1:100, mouse anti-HSV(1+2) gB, ab6506, Abcam, Cambridge, UK) were incubated on the glass slides at 4°C overnight, then detected with red fluorescently-labeled goat-anti-mouse secondary antibodies (BA1031, Boster company, Wuhan, China) at 37°C for an hour in the dark. Both nuclei were performed counterstain with 0.5 ug/ml DAPI (Beyotime Biotechnology, Shanghai, China) for 10 mins at room temperature. All the coverslips were mounted on the glass slides using anti-quenching Fluoroshield™ histology mounting medium (Sigma-Aldrich, USA) and visualized under a fluorescence microscope (BX51TF, Olympus company, Tokyo, Japan) with magnification 10 × or 20×.

### DAB staining (Amplifier Polymer)

The DAB staining was performed using a commercial kit called DAB Detection Kit (EliVision Super DAB, Maixin biotech company, Fuzhou, China). After paraffin sections of vaginal tissues as well as 3D cultures solvent-dewaxing and treatment with microwave antigen retrieval, endogenic peroxidase blocker were added at room temperature for 10 mins and the primary antibodies mouse anti-p63 (1:100, ab735, Abcam, Cambridge, UK), rabbit anti-Laminin (1:100, ab11575, Abcam, Cambridge, UK), mouse anti-CK18 (1:100, sc-32329, Santa Cruz Biotechnology, CA, USA), mouse anti-CK14 (1:100, sc-23878, Santa Cruz Biotechnology, CA, USA), mouse anti-E-cadherin (1:100, sc-71008, Santa Cruz Biotechnology, CA, USA), mouse anti-Dsg-1 (1:100, sc-13716, Santa Cruz Biotechnology, CA, USA) were respectively incubated on the slides at room temperature for an hour. The slides were then added the reaction-amplified reagent for 20 mins and conjugated with high-sensibility enzyme-conjugated lgG polymer. Reactants were visualized with the fresh-prepared DAB chromogenic solutions for 3 to 5 mins. Hematoxylin somatic cell staining reagent was used to counterstain nuclei for 8 mins. Glass slides were finally mounted with neutral balsam and visualized under EVOS visual imaging microscope (Life Technologies Corp Bothell, WA, USA).

### Hematoxylin and Eeosin (H&E) staining

Normal vaginal tissues and 3D cultures were fixed in 4% paraformaldehyde, dehydrated through a stepwise series of ethanol organic solutions and paraffin-embedded according to standard histological procedure [48]. Paraffin blocks were then successively sectioned in 5 μm thickness and mounted on the glass slides. The paraffin-embedded sections were stained with Hematoxylin and Eeosin (H&E) (Zhongshan Golden Bridge Company, Beijing, China). Morphological observation of H&E-stained vaginal tissues and 3D cultures were photographed under the EVOS visual imaging microscope (Life Technologies Corp Bothell, WA, USA).

### Soft agar assay

Three microliter 0.6% low-melting point agarose (Sigma-Aldrich, Louis, MO, USA) in culture medium containing 10% fetal bovine serum (FBS) and 1% antibiotics (penicillin/streptomycin, amphotericin) was poured into a 6-well plate (three wells per group) and allowed to solidification at room temperature after 1 hr. After solidity, 3 × 10^4^ cells were suspended in 500 μl culture medium into 2 ml 0.3% low-melting point agarose and placed on top of the bottom layer. All the cells were grown at 37°C for 3–4 weeks. Colonies were visualized and photographed under the visual imaging microscope. The ability of forming colonies of different cells were counted.

### Total RNA extraction and taqman assay

hTERT mRNA expression in HNVEC cells was assayed by quantitative real-time PCR. Total RNA was extracted with TRIzol^®^ reagent (Invitrogen, USA) according to the manufacturer's specifications. The purity of RNA was determined according to denaturing gel electrophoresis and identified by the ratio of protein and nucleic acid optical density for 1.8 to 2.0. Two microgram of total RNA was reverse transcribed to cDNA using a High Capacity cDNA Reverse Transcription Kit (2090B, Takara Biotechnology Co., Ltd, Dalian, China). Amplification of human β-actin mRNA was an internal control to normalized the amount of samples added to the reaction. The first strand cDNA was used as a template on the basis of TaqMan probe method to achieve PCR amplification on a CFX96^™^ Real-Time System (Bio-rad, California, CA, USA) using fast mode. The real-time PCR procedure was set, and hTERT mRNA was measured using the methods and primers that have been previously published [18, 49, 50].

## SUPPLEMENTARY MATERIALS FIGURES AND TABLES


