# Supplementary material for: Ex vivo 2D and 3D HSV-2 infection model using human normal vaginal epithelial cells
Source: Oncotarget. 2017 Jan 27;8(9):15267–82. doi: 10.18632/oncotarget.14840 (PMC5362485; doi:10.18632/oncotarget.14840)
Supplement: Supplementary file 1 [file oncotarget-08-15267-s001.pdf]

Ex vivo 2D and 3D HSV-2 infection model using human normal vaginal epithelial cells

Supplementary Materials

A

STR sites	Allele 1	Allele2
Amel	X	
CSF1PO	11	12
D10S1248	13	14
D12S391	19	22
D13S317	12	14
D16S539	9	
D18S51	13	18
D19S433	13	14
D21S11	30	31.2
D2S1338	24	
D2S441	9.1	11
D3S1358	15	
D5S818	10	12
D6S1043	18	20.3
D7S820	8	12
D8S1179	13	14
FGA	21	24
Penta D	9	
Penta E	14	16
TH01	9	
TPOX	8	11
vWA	14	

B

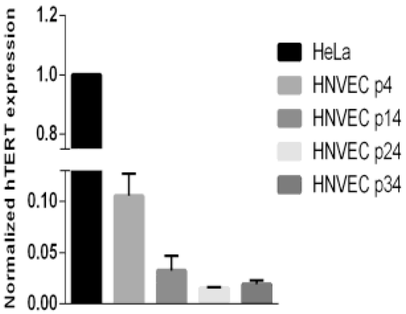

Supplementary Figure 1: STR analysis and hTERT expression of human normal vaginal epithelial cells (HNVEC). (**A**) The Short Tandem Repeat (STR) Analysis of HNVEC cells. HNVEC cells have 21 STR loci and a couple of X-chromosome-specific Amelogenin loci that do not match any other cell lines registered or published before. (**B**) Expression of hTERT in HNVEC cells. Quantitative real-time PCR of hTERT expression in HNVEC cells was performed at the indicated passages (p4, p14, p24 and p34). HeLa cells were used as control.

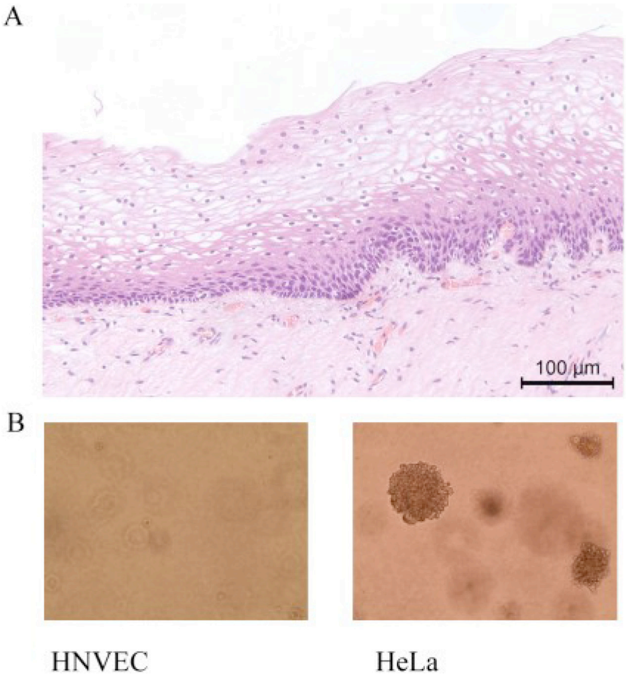

Supplementary Figure 2: Normal tissue origin and non-oncogenicity of HNVEC. (**A**) H&E-staining histological section of original vaginal tissue. The vaginal tissue was fixed by 4% paraformaldehyde (wt/vol), and then paraffin-embedded and sectioned using standard histological procedures. Magnification 20×. (**B**) HNVEC cells do not form colonies in soft agar. The tumorigenicity and colony-forming ability of HNVEC cells was evaluated by soft agar assay. The cell colonies were observed and photographed after 30 days of culture. HeLa is the control cell line. Magnification 10×.

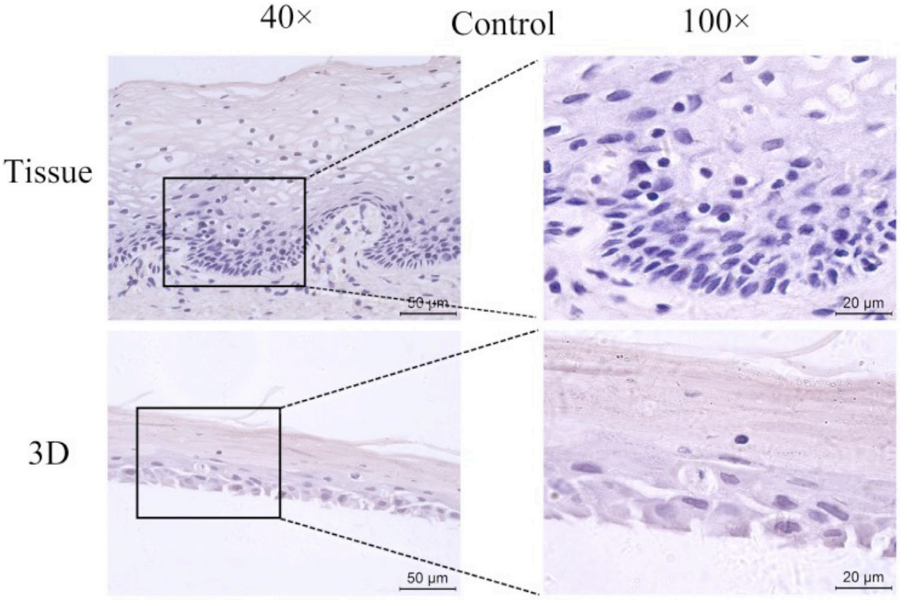

Supplementary Figure 3: The negative control of immunohistochemical staining. The HNVEC cells were cultured in air-liquid interface (ALI) for 14 days before fixed. The originated human vaginal tissue or 3D cultures were fixed by 4% paraformaldehyde (w/v), and paraffin-embedded, sectioned, stained without primary anitibody. Scale bar, 50 µm and 20 µm.
